# Signature of circular RNAs in human induced pluripotent stem cells and derived cardiomyocytes

**DOI:** 10.1186/s13287-018-0793-5

**Published:** 2018-03-09

**Authors:** Wei Lei, Tingting Feng, Xing Fang, You Yu, Junjie Yang, Zhen-Ao Zhao, Junwei Liu, Zhenya Shen, Wenbo Deng, Shijun Hu

**Affiliations:** 1 Institute for Cardiovascular Science & Department of Cardiovascular Surgery of the First Affiliated Hospital, Soochow University, Suzhou, China; 2 Key Laboratory of Stem Cells and Biomedical Materials of Jiangsu Province and Chinese Ministry of Science and Technology, Soochow University, Suzhou, China; 3 Department of Cardiovascular Surgery, Union Hospital, Tongji Medical College, Huazhong University of Science and Technology, Wuhan, China; 4 Division of Reproductive Sciences, Cincinnati Children’s Hospital Medical Center, Cincinnati, OH USA

**Keywords:** Induced pluripotent stem cell, Cardiomyocyte, Circular RNA

## Abstract

**Background:**

Circular RNAs (circRNAs) are regarded as a novel class of noncoding RNA regulators. Although a number of circRNAs have been identified by bioinformatics analysis of RNA-seq data, tissue and disease-specific circRNAs are still to be uncovered to promote their application in basic research and clinical practice. The purpose of this study was to explore the circRNA profiles in human induced pluripotent stem cells (hiPSCs) and hiPSC-derived cardiomyocytes (hiPSC-CMs), and to identify cardiac or disease-specific circRNAs.

**Methods:**

hiPSCs were generated from fibroblasts, and then further differentiated to hiPSC-CMs by modulating WNT signaling in RPMI+B27 medium. Following high-throughput RNA sequencing, circRNAs were extracted and quantified by a combined strategy known as CIRCexplorer. Integrative analysis was performed to illuminate the correlation between circRNAs and their parental linear isoforms. Cardiac and disease-specific expression of circRNAs was confirmed by quantitative reverse-transcription PCR.

**Results:**

In this study, a total of 5602 circRNAs were identified in hiPSCs and hiPSC-CMs. Our data indicated, for the first time, more enriched expression of circRNAs in differentiated cardiomyocytes than in undifferentiated hiPSCs. In addition to the host gene-dependent expression, our integrative analysis also identified a number of circRNAs showing host gene-independent expression in hiPSCs and hiPSC-CMs. CircRNAs including *circSLC8A1*, *circCACNA1D*, *circSPHKAP* and *circALPK2* showed cardiac-selective expression during cardiac differentiation and human heart-specific enrichment in fetal tissues. Furthermore, *circSLC8A1* abnormally increased in heart tissues from patients suffering from dilated cardiomyopathy.

**Conclusions:**

CircRNAs are highly enriched in hiPSC-differentiated CMs, and cardiac-specific circRNAs such as *circSLC8A1*, *circCACNA1D*, *circSPHKAP* and *circALPK2* may serve as biomarkers of CMs. Detection of the excessive expression of *circSLC8A1* provides a potential approach for pathological status indication of heart disease.

**Electronic supplementary material:**

The online version of this article (10.1186/s13287-018-0793-5) contains supplementary material, which is available to authorized users.

## Background

Heart diseases have become the leading cause of morbidity and mortality in the world [1]. Conventional treatments are suboptimal for heart disease caused by ischemia-induced irreversible death of heart muscle. Since the endogenous capacity of regeneration is insufficient to remedy the injured myocardium, stem cell transplantation is being widely regarded as the most attractive approach for patients with heart diseases [2]. The advent of human embryonic stem cells (ESCs) and induced pluripotent stem cells (iPSCs) provided ever more suitable cell sources for repairing damaged myocardium in human. Bypassing ethical issues, iPSCs are believed more readily available for cellular transplantation, especially in personalized therapies [3]. Recent studies have reported several similar methods of cardiomyocyte differentiation from human ESCs and iPSCs using chemically defined systems, and these pluripotent stem cell (PSC)-derived cardiomyocytes (hPSC-CMs) were proven to enhance cardiac function by integrating into infarcted hearts in large animal models of myocardial infarction [4–6]. However, hPSC-CMs generated by current methods were heterogeneous populations containing non-CM cells, and not ready for clinical use. In order to improve the technology of CM differentiation, scientists urgently need to illuminate the underlying molecular mechanisms driving cardiac fate, and search for reliable biomarkers.

In addition to protein-coding genes, the participation of noncoding RNAs (ncRNAs), including miRNAs and long ncRNAs, highly raised the complexity of the regulatory network in cardiovascular differentiation, heart development and cardiovascular pathophysiology [7–10]. Due to the development of high-throughput sequencing and bioinformatics analysis, abundant circular RNAs (circRNAs), a novel class of ncRNA, were recently discovered in mammals, and showed a tissue-specific expression in the heart, brain, liver and so on [11]. CircRNAs form a covalently closed continuous loop by back-splicing of the 3′-end to 5′-end of the same or an upstream exon. Despite the controversy, circRNAs are classified as a type of noncoding RNA, as they lack the 5′-cap structure requisite for canonical ribosome-based translation, and little evidence was reported for circRNA-coding proteins in cells [12]. Several studies have demonstrated functions of circRNAs as cis regulators of transcription and sponges of miRNAs or RNA binding proteins [13, 14]. However, the biological functions and mechanisms of most circRNAs are largely unknown.

Several RNA-sequencing analyses have revealed the widespread expression of circRNAs in heart development and heart diseases [15–18]. Among these studies, Tan et al. [18] and Werfel et al. [15] each identified a large number of circRNA candidates in the human heart. Some circRNAs have been uncovered in PSCs and their derived CMs [18, 19]. Multiple circRNAs such as circular isoforms of genes have also been reported to be differentially expressed in human diseased hearts compared to healthy normal controls [15, 16]. Due to the wide variation of circRNAs, more high-throughput analyses and detailed research are necessary to discover reliable circRNA biomarkers in cardiac differentiation and heart diseases.

In this study, we characterized 5602 circRNAs in hiPSCs generated from fibroblasts and hiPSC-CMs, and detected a high enrichment of circRNAs in differentiated hiPSC-CMs. The integrative analysis identified a number of circRNAs showing host gene-independent expression. Differentially expressed circRNAs were validated by reverse-transcription polymerase chain reaction (RT-PCR). Stage-specific expression of circRNAs was further investigated across cardiac differentiation from hiPSCs. We finally explored the possibility of using circRNAs as biomarkers of heart diseases.

## Methods

### Human tissue collection

Experiments with donated human adult and fetal tissues were approved by the ethical committees of Soochow University, Suzhou, China and Huazhong University of Science and Technology, Wuhan, China. After informed consent, normal and diseased adult heart tissues were taken from healthy individuals or DCM patients receiving cardiac transplantation, respectively. Fetal samples such as the brain, heart, liver, spine and stomach were harvested from abortions aged between 9 and 10 weeks post gestation, with informed consent from the parents.

### Isolation of fibroblasts and hiPSC generation

Skin biopsies were minced and digested in collagenase IV (Thermo Fisher Scientific Inc., MA, USA) in Dulbecco’s modified Eagle medium (DMEM; Gibco) at 37 °C for 6 h. After passing through a nylon mesh, dispersed dermal fibroblasts were washed three times with PBS, and cultured in DMEM supplemented with 10% fetal bovine serum (FBS; Gibco) at 37 °C in a humidified incubator with 5% CO_2_ and 95% air. Fibroblasts within five passages were plated on gelatin-coated dishes in DMEM with 10% FBS, and subsequently infected by lentiviral particles encoding four stem cell factors (*OCT4*, *SOX2*, *KLF4* and *cMYC*). Five days later, infected cells were passaged onto irradiated MEF feeder layers, and further maintained in KSR medium (DMEM/F12 supplemented with 20% KnockOut Serum Replacement, 4 ng/ml bFGF, 2 mM l-glutamine, 1% MEM-non essential amino acids, and 0.007% 2-mercaptoethanol). On days 20–30 post infection, individual colonies with human ESC-like morphology were picked and maintained on Matrigel-coated dishes in E8 media. Colonies with 75–90% confluence were passaged by 5-min incubation with 0.5 mM EDTA in DPBS without Ca^2+^ and Mg^2+^.

### Cardiac differentiation from hiPSCs

Monolayer-directed differentiation of hiPSC into CMs was performed as described previously with slight modifications [20]. Four days before initiation of cardiac differentiation, hiPSCs were split in a 1:8 ratio. On day 0 of cardiac differentiation, hiPSCs with 90% confluence were incubated with RPMI-B27 without insulin (Life Technologies, CA, USA) supplemented with 6 μM CHIR99021 (LC Laboratories, MA, USA) for 48 h. After withdrawn of CHIR99021, cells were kept in RPMI-B27 without insulin for another 48 h, and followed by IWR1 (LC Laboratories) treatment in the same basal medium for 48 h. Media were then switched to RPMI-B27 supplemented with insulin for further culturing. Beating hiPSC-CMs were expected to be observed from day 9 post initiation of cardiac differentiation. On day 11, derived hiPSC-CMs were metabolically purified by 4-day culture in RPMI-B27 without d-glucose, and then maintained in RPMI-B27 supplemented with insulin.

### Identification of hiPSCs and hiPSC-CMs

The pluripotency of hiPSCs was verified by the methods of embryoid body (EB) and teratoma formation, as well as immunofluorescence staining of stem cell markers including OCT4, NANOG and TRA-1-60 as described previously [21]. The hiPSC-CMs were identified by morphological analysis, calcium transient analysis and immunofluorescence staining of cardiac markers including TNNT2 and α-SA as described previously [22].

### RNA sequencing

Total RNAs from hiPSCs and their derived iPSC-CMs on day 30 were extracted using the RNeasy kit (Qiagen, CA, USA), and treated with DNase I (Qiagen). Libraries for RNA sequencing were generated using Ovation® RNA-Seq System V2 (NuGEN, CA, USA), as per the manufacturer’s instructions. Barcoded libraries were then prepared using the NEBNext® DNA Library Prep Master Mix Set for Illumina®, followed by gel excision and extraction to collect the 250-bp fragments. Fragments were sequenced on the HiSeq2000 platform using paired-end reads, with an average of 30 million reads per sample. Sequence reads were first multiply mapped against the GRCh37/hg19 human reference genome using TopHat 2.1. Unmapped reads were then extracted and mapped onto the relevant reference genome using TopHat-Fusion. Reads that split and aligned on the same chromosome but in noncollinear ordering were extracted as candidate back-spliced junction reads. Back-spliced junction reads were further realigned against existing gene annotations to determine the precise positions of donor or acceptor splice sites for each back-spliced event. Finally, back-spliced junction reads were combined and scaled to RPB (reads per billion mapped reads, including TopHat mapping and TopHat-Fusion mapping) to quantify every back-spliced event.

### Gene Ontology

Metascape (http://metascape.org) was applied for Gene Ontology (GO) term detection and clustering.

### *QKI* knockdown by siRNA transfection

Three types of siRNAs targeting the human *QKI* gene were designed and synthesized by Guangzhou RiboBio Co. (China), and then transfected into hiPSC-CMs with Lipofectamine 2000 (Invitrogen) according to the manufacturer’s instructions. Two of the three siRNAs showed significant inhibitory effects on *QKI* expression in hiPSC-CMs, and thus were used for subsequent studies.

### RT-PCR and quantitative real-time PCR

RNAs were subjected to reverse transcription with the PrimeScript reverse transcriptase reagent kit (TaKaRa, Dalian, China). PCR was performed using divergent primers to amplify only circular transcripts. Amplified fragments were visualized by gel electrophoresis. All primers used in this study are presented in Additional file 1: Table S1. The PCR products were further confirmed by Sanger sequencing. Quantitative real-time PCR (qRT-PCR) was performed using a SYBR Premix Ex Taq kit (TaKaRa) on the StepOnePlus Real-Time PCR System (Applied Biosystems™, CA, USA). Reactions were run in triplicate for at least three biological replicates. Data were normalized to the average expression of 18S rRNA, and analyzed using 2^–ΔΔCT^ method.

### Statistical analysis

Statistical analysis of the qRT-PCR results was performed using Student’s *t* test or one-way ANOVA as detailed in the figure legends. *p* < 0.05 was considered statistical significance. Data are presented as mean ± SD.

## Results

### Identifying and characterizing circRNAs in hiPSCs and hiPSC-derived cardiomyocytes

**Fig. 1:**
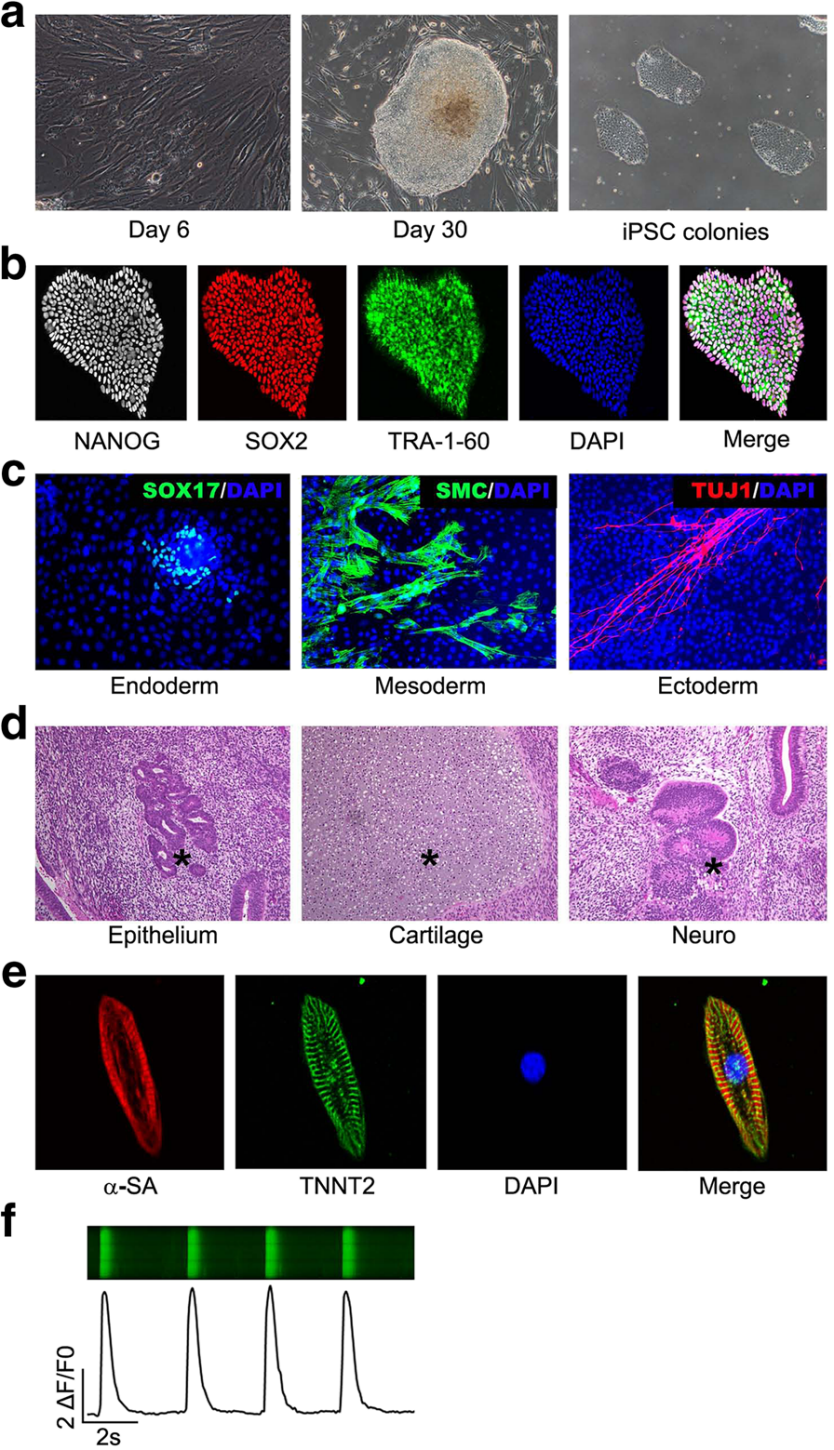
Identification of hiPSCs and hiPSC-derived cardiomyocytes. **a** Representative photographs of fibroblasts on day 6 and human fibroblast-derived iPSC colonies on day 30 during hiPSC generation, as well as passaged hiPSCs under bright field at 100× magnification. **b** Immunofluorescence staining of hiPSCs for pluripotency markers including NANOG, SOX2 and TRA-1-60, and nucleus marker DAPI. **c** Immunofluorescence staining of hiPSC-derived cell for markers of endoderm (SOX17), mesoderm (SMC), ectoderm (TUJ1) and nucleus (DAPI). **d** Hematoxylin and eosin staining of hiPSC-derived teratoma comprising of epithelium, cartilage and neuro (asterisks). **e** Immunofluorescence staining of hiPSC-CMs for cardiac markers including α-SA and TNNT2, and nucleus marker DAPI. **f** Line-scan images and corresponding spontaneous Ca^2+^ transients in hiPSC-CMs. Ca^2+^ level calculated as ΔF/F0 = (F – F0) / F0, where F0 is diastolic fluorescence level. iPSC induced pluripotent stem cell

Reprogramming of fibroblasts into hiPSCs was initiated by lentiviral-mediated overexpression of four key transcription factors (*OCT4*, *KLF4*, *c-MYC* and *SOX2*), as described previously [23]. The isolated hiPSC colonies were cultured in E8 culture medium (Fig. 1a), and further verified by expression of pluripotency markers NANOG, SOX2, OCT4 and TRA-1-60 (Fig. 1b and Additional file 2: Figure S1A), and formation of embryoid bodies (EBs) (Additional file 2: Figure S1B) and teratoma with three germ layers (Fig. 1c, d). Human iPSCs were then further differentiated to CMs by modulating WNT signaling in RPMI+B27 medium. Biomarker genes of early mesoderm (*BRA*), cardiogenic mesoderm (*MESP1*), cardiac-specific progenitors (*TBX5*) and structural genes of CMs (*TNNT2*, *MYH6* and *MYH7*) displayed a stage-specific expression in hiPSC-derived cells during CM differentiation (Additional file 2: Figure S1C–H). These hiPSC-derived CMs (hiPSC-CMs) exhibited spontaneous beating (Additional file 3: Video 1), high expression of cardiac marker genes such as TNNT2 and sarcomeric α-actinin (α-SA) as shown in Fig. 1e, and typical calcium transient (Fig. 1f). These data indicate successful generation of hiPSCs and cardiac differentiation from hiPSCs.

**Fig. 2:**
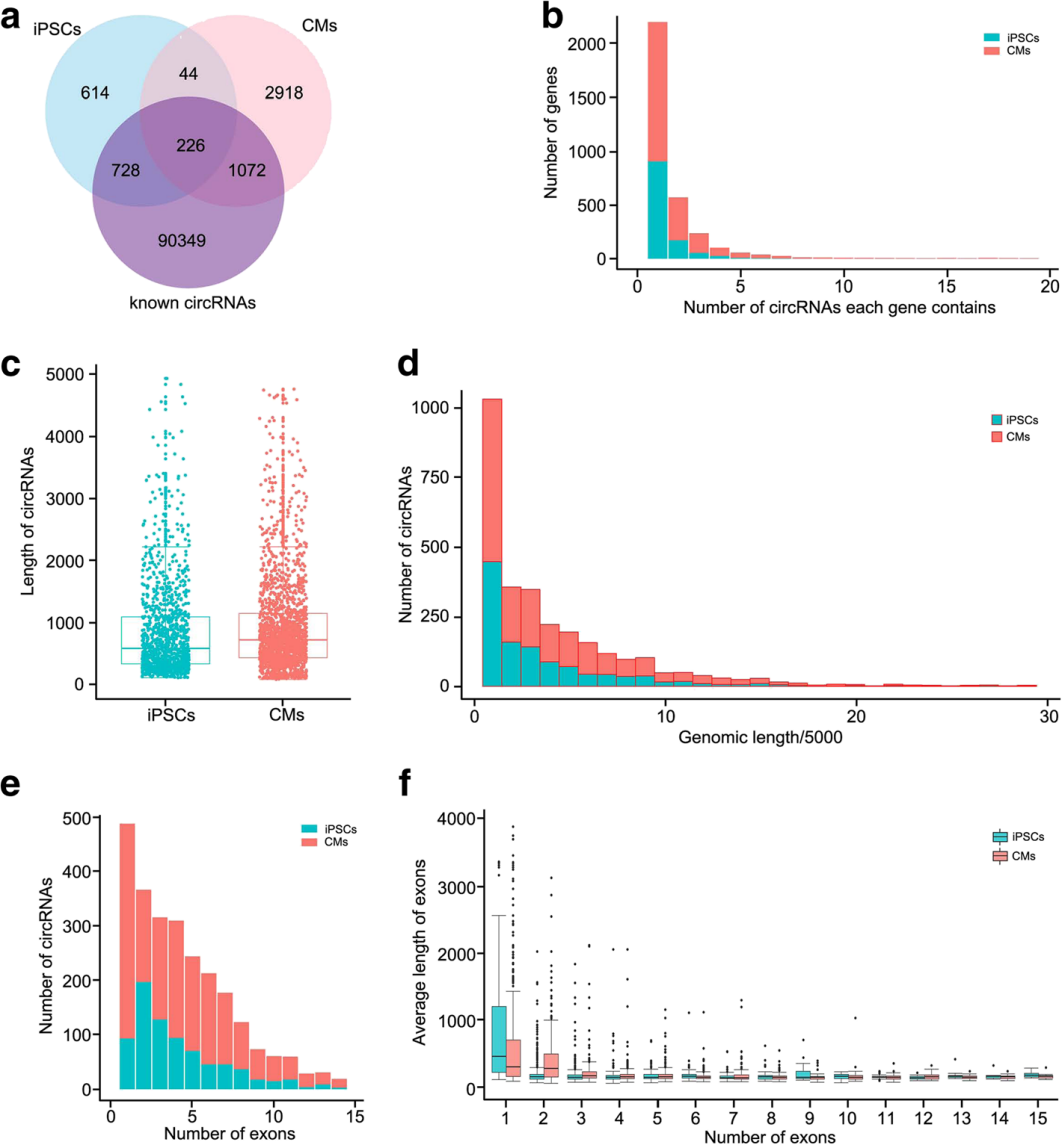
Genomic features of circRNAs in hiPSCs and hiPSC-CMs. **a** Venn diagram illustrating abundant novel circRNAs in hiPSC (iPSC) and hiPSC-CM (CM) samples. **b** Most genes in both hiPSCs and hiPSC-CMs generated only one circular isoform. **c** No significant difference in length of circRNAs observed between hiPSCs and hiPSC-CMs. Most circRNAs spanned less than 45 kb of genomic DNA (**d**), and were composed of one to eight exons (**e**). **f** Average length of exons was larger in single-exon circRNAs than in multiple-exon circRNAs. circRNA circular RNA, CM cardiomyocyte, iPSC induced pluripotent stem cell

RNAs from hiPSCs and hiPSC-CMs (passage 30) were then subjected to RNA sequencing, and back-spliced reads in RNA-sequencing data were extracted, annotated and quantified by a combined strategy known as CIRCexplorer with default parameters, and finally scaled to RPB (reads per billion mapped reads). Only back-spliced junctions covered by at least two unique reads in a single sample were selected for further analysis. We totally identified 5602 circRNAs, including 1612 in the hiPSC group and 4260 in the hiPSC-CM group with 270 in common. Among these, more than 3500 circRNAs were not yet included in circBase, a database for circRNAs (Fig. 2a) [24]. Furthermore, there were 614 and 2918 new circRNAs exclusively expressed in hiPSCs and hiPSC-CMs, respectively, indicating the cell type specificity of circRNAs (Fig. 2a). All of the identified circRNAs were summarized in Additional file 4: Table S2.

We then investigated the genomic features of circRNA. While the majority of host genes generated one to seven circRNA isoforms, the number of host genes considerably decreased along with the increasing number of circular isoforms in both the hiPSC and hiPSC-CM groups (Fig. 2b). Compared to the hiPSC group, genes enriched in hiPSC-CMs generated more circular isoforms (Fig. 2b). For example, we totally identified 129 different circRNA isoforms of Titin (*TTN*), a gene containing the largest number of exons and encoding a large abundant protein of striated muscle (Additional file 4: Table S2). However, no circular isoform of pluripotent genes such as *OCT4* and *NANOG* was detected in hiPSCs. Most circRNAs had a length ranging from 200 to 2500 nucleotides (Fig. 2c), and spanned a genomic distance less than 45 kb in both groups (Fig. 2d). While circRNAs, either in hiPSCs or hiPSC-CMs, usually contained one to eight exons (Fig. 2e), the exon length of single-exon circRNAs was much longer than the average exon length of circRNAs with multiple exons (Fig. 2f).

### CircRNAs are highly enriched in hiPSC-derived cardiomyocytes

**Fig. 3:**
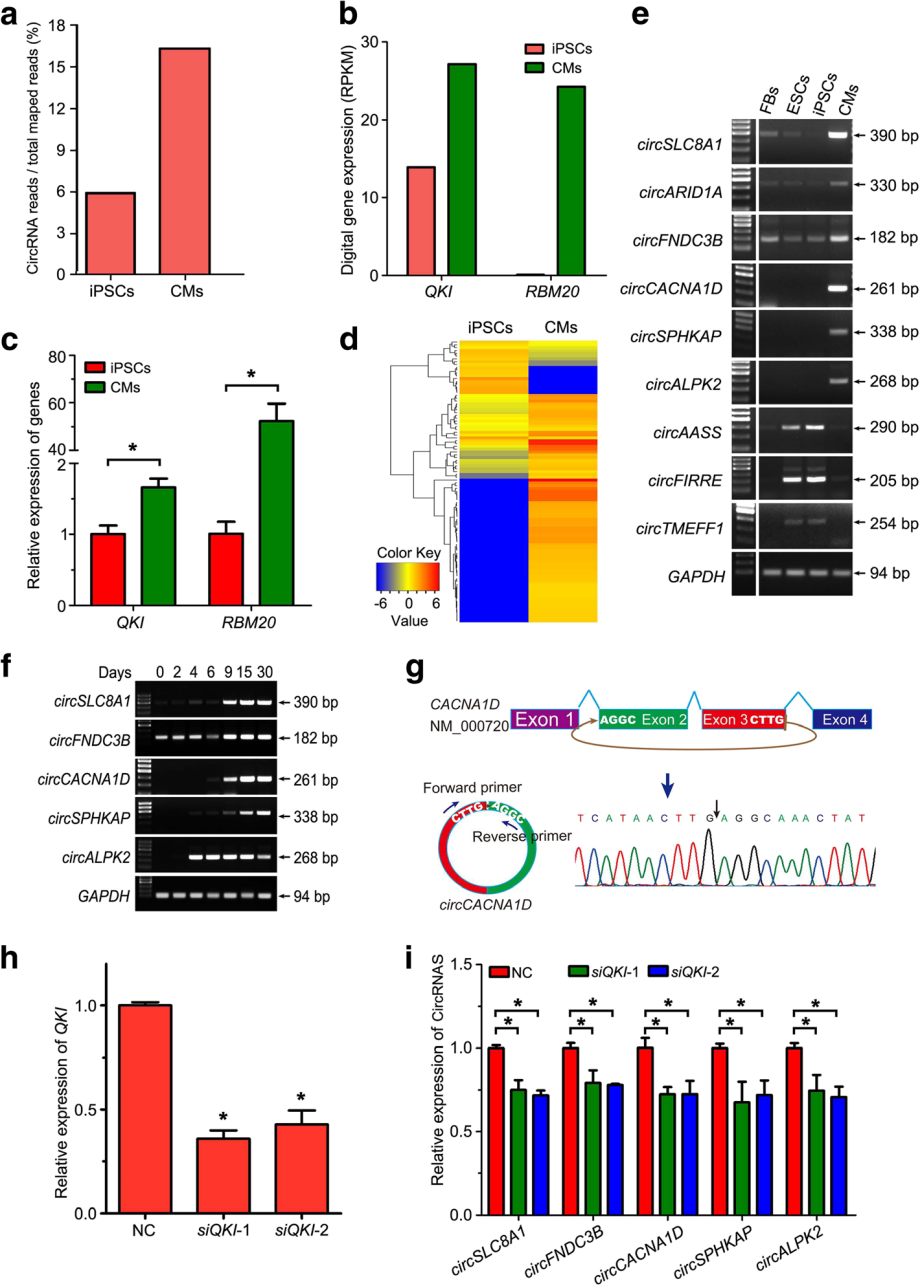
Cell-specific expression of circRNAs in hiPSCs and hiPSC-CMs. **a** Proportion of circRNA reads among total mapped reads higher in the hiPSC-CM group (CM) than in the hiPSC group (iPSC). **b** RNA-seq data showed increased expression of Quaking (*QKI*) and RNA binding Motif protein 20 (*RBM20*), two alternative factors associated with circRNA formation. **c** qRT-PCR detection of *QKI* and *RBM20* mRNA in hiPSCs and hiPSC-CMs; data shown as mean ± SD, Student’s t test, *p < 0.05. **d** Hierarchical clustering (heat map) showed differential expression of circRNAs among hiPSCs and hiPSC-CMs. **e** Confirmation of differentially expressed circRNAs by RT-PCR. **f** Representative electrophoretograms of RT-PCR showed differential expression of circRNAs from days 0 to 30 during cardiac differentiation from hiPSCs. **g** Schematic depiction of *circCACNA1D* generation by back-splicing of exon 3 to exon 2, and verification of back-splicing site of *circCACNA1D* by Sanger sequencing. **h** qRT-PCR detection of *QKI* mRNA in negative control (NC) and *siQKI* transfected hiPSC-CMs. **i** qRT-PCR showed decreased expression of circRNAs in *siQKI* transfected hiPSC-CMs, compared with that in NC. circRNA circular RNA, CM cardiomyocyte, ESC embryonic stem cell, FB fibroblast, iPSC induced pluripotent stem cell. Data in **h** and **i** shown as mean ± SD, one-way ANOVA followed by Turkey’s test, *p < 0.05

As shown in Fig. 2a, many more circRNAs were identified in hiPSC-CMs rather than hiPSCs. We thus calculated the ratio of circRNA reads in total mapped reads in both samples, and observed a significant increase of circRNA reads in hiPSC-CMs compared to that in the hiPSC sample (17.5% vs 6%) (Fig. 3a). Both results suggested the enrichment of circRNAs in differentiated CMs compared to undifferentiated hiPSCs. Correspondingly, our RNA-sequencing data also revealed a substantial increase of two alternative factors associated with circRNA formation, Quaking (*QKI*) and RNA binding Motif protein 20 (*RBM20*), in hiPSC-CMs when compared with that in hiPSCs (Fig. 3b). The increased expression of *QKI* and *RBM20* mRNA in hiPSC-CMs was further verified by our qPCR results (Fig. 3c).

A heat map of the top 100 circRNAs indicated cell-specific expression of circRNAs in hiPSCs and hiPSC-CMs (Fig. 3d). More circRNAs were specifically expressed in hiPSC-CMs and only few circRNAs showed a decreased expression in the hiPSC-CM group when compared with the hiPSC group. We further performed RT-PCR to verify these differentially expressed circRNAs, using divergent primers to amplify only circular transcripts but not linear transcripts. Consistent with our algorithms, circRNAs generated from exons of six protein-coding genes (*SLC8A1*, *ARID1A*, *FNDC3B*, *CACNA1D*, *SPHKAP* and *ALPK2*) were highly enriched in hiPSC-derived CMs, while the expression levels of *circAASS*, *circFIRRE* and *circTMEFF1* were obviously decreased in hiPSC-CMs and fibroblasts compared to both hESC and hiPSC groups (Fig. 3e). The cardiac expression of *circSLC8A1*, *circCACNA1D*, *circSPHKAP* and *circALPK2* suggest that these circRNAs could be potential biomarkers for cardiomyocytes.

To further verify these cardiac biomarkers, RNAs isolated from cells in different stages of cardiomyocyte differentiation were subjected to RT-PCR analysis. As shown in Fig. 3f, while abundant expression of *circALPK2* was observed in cells on day 4 onward, *circSLC8A1*, *circCACNA1D* and *circSPHKAP* RNAs were highly expressed in the beating cardiomyocytes on days 9, 15 and 30 of cardiac differentiation. In spite of its wide expression in all stages, *circFNDC3B* transcripts were obviously increased in differentiated cardiomyocytes from day 9. Subsequently, we verified the amplified product of RT-PCR by Sanger sequencing as shown in Fig. 3g and Additional file 5: Figure S2. These data suggested that *circSLC8A1*, *circCACNA1D* and *circSPHKAP* may serve as novel biomarkers during cardiac differentiation from hESCs or hiPSCs.

We then attended to exploring the role of alternative factor Quaking in circRNA expression in hiPSC-CMs. As shown in Fig. 3h, i, siRNA-mediated inhibition of *QKI* expression significantly downregulated the expression levels of circRNAs including *circSLC8A1*, *circFNDC3B*, *circCACNA1D*, *circSPHKAP* and *circALPK2* in hiPSC-CMs. These results indicated that enrichment of circRNAs in differentiated hiPSC-CMs might be particularly associated with the increased expression of alternative factors such as *QKI*.

### Integrative analysis of circRNAs and host gene expression data

Since circRNAs are generated by back-splicing of exons of parental transcripts, we performed correlation analysis based on our RNA-sequencing data. Firstly, we analyzed the linear transcriptome in hiPSCs and hiPSC-CMs. Based on the cutoff value at 50 RPKM, we identified a total of 1270 differentially expressed genes with at least 2-fold change in hiPSC-CMs, compared to hiPSCs (Additional file 6: Figure S3A, Additional file 7: Table S3). Pluripotency genes such as *NANOG*, *SOX2* and *OCT4* (also known as *POU5F1*) were among the downregulated genes (Additional file 6: Figure S3A, Additional file 7: Table S3). On the contrary, the expression of structural genes encoding for sarcomeric-related proteins of terminal differentiated cardiomyocytes, including *TNNT2*, *MYL2, MYH6*, and *MYH7*, were significantly elevated in the hiPSC-CM group (Additional file 6: Figure S3A, Additional file 7: Table S3). Gene Ontology (GO) analysis revealed that genes upregulated in hiPSCs were enriched for the cell cycle, transcriptional regulation of PSCs, DNA replication and chromatin remodeling at the centromere (Additional file 6: Figure S3C, E), while genes upregulated in hiPSC-CMs were involved in heart development, muscle system process, striated muscle cell differentiation, and so on (Additional file 6: Figure S3D, F). These bioinformatics results demonstrate the reliability of our data.

**Fig. 4:**
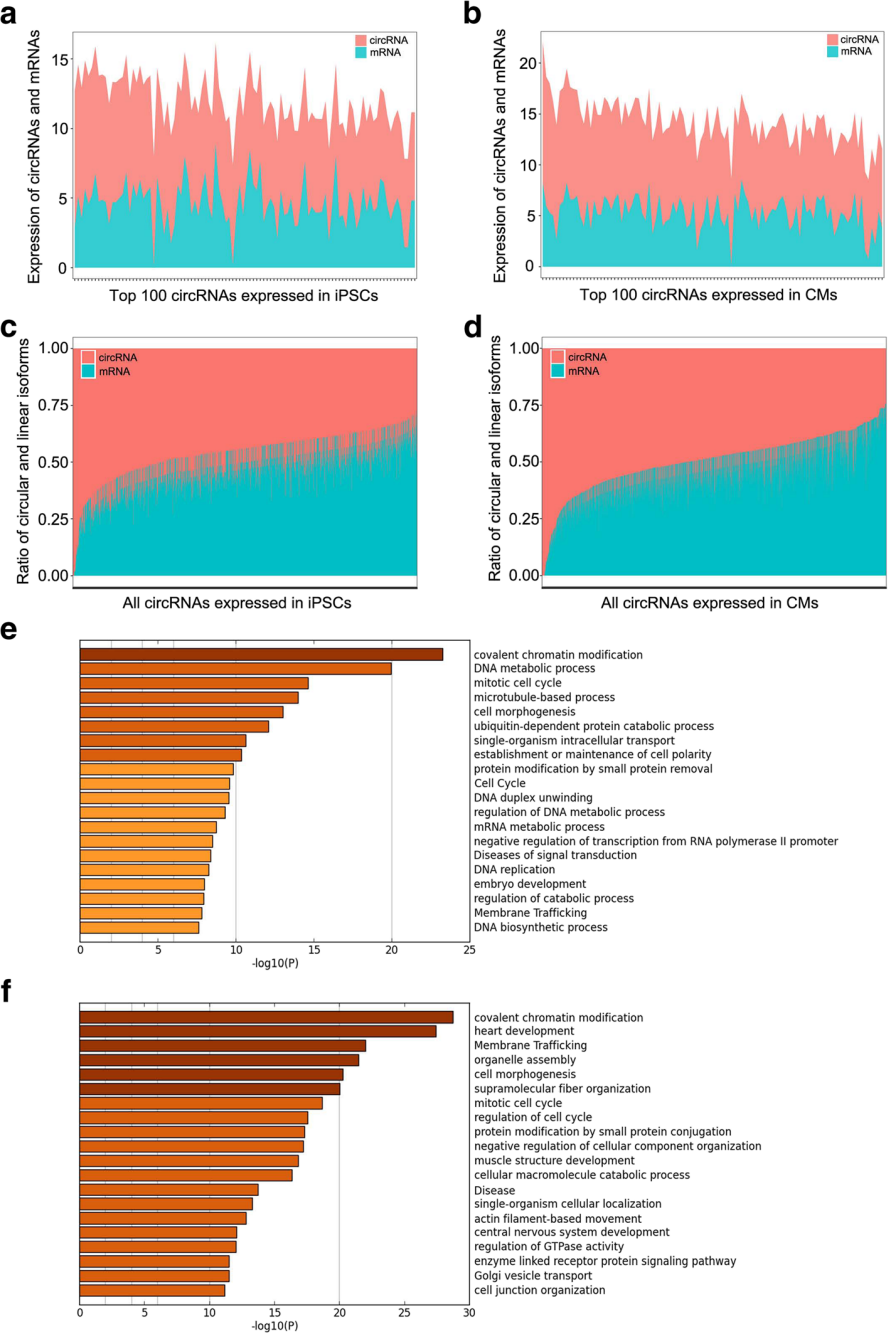
Integrative analysis of circRNAs and their parental linear transcripts. **a**, **b** Relationship between top 100 circRNAs and their parental linear transcripts in hiPSCs (iPSC) and hiPSC-CMs (CM). Ratio of circular and linear isoforms of each gene expressed in hiPSCs (**c**) and hiPSC-CMs (**d**) were converted from the relative value of circRNA (RPB)/mRNA (RPKM). Based on cutoff value for proportion of circRNAs at 75%, many more circRNAs showed a host gene-independent expression in hiPSC-CMs, compared with that in hiPSCs. Gene Ontology analyses of host genes of upregulated circRNAs in hiPSCs (**e**) and hiPSC-CMs (**f**) indicated most enriched 20 signal pathways with *p* < 10^−5^. circRNA circular RNA, CM cardiomyocyte, iPSC induced pluripotent stem cell

The following correlation analysis demonstrated a strong positive correlation of expression between the top 100 circRNAs and their linear parental transcripts in both hiPSCs and hiPSC-CMs (Fig. 4a, b). In order to seek the host gene-independently expressed circRNAs, we calculated the ratio of circular and linear isoforms of each gene. As shown in Fig. 4c, d, the proportion of most circRNAs is approximately 50%. Interestingly, we also found some circRNAs exhibiting distinguish expression patterns with their linear parental transcripts, based on the cutoff value of the proportion of circRNAs at 75% (Fig. 4c, d). It is worth noting that more circRNAs revealed a host gene-independent expression in the hiPSC-CM group, when compared with that in the hiPSC group. Additional file 8: Table S4 presents the enriched and host gene-independently expressed circRNAs in hiPSCs and hiPSC-CMs.

The parental genes of differentially expressed circRNAs were then subjected to Gene Ontology. As shown in Fig. 4e, f, both hiPSCs and hiPSC-CMs expressed abundant circRNAs transcribed from genes associated with same categories, such as covalent chromatin modification, cell cycle and cell morphogenesis, but belong to different subclasses. Furthermore, host genes of upregulated circRNAs in CMs were also enriched for myocardial function such as heart development, membrane trafficking, organelle assembly, fiber organization and muscle structure development (Fig. 4f). Instead, genes generating hiPSC-specific circRNAs were enriched in the pathway of DNA metabolic process, DNA replication and so on (Fig. 4e).

### Differential expression of circRNAs in human healthy and diseased hearts

**Fig. 5:**
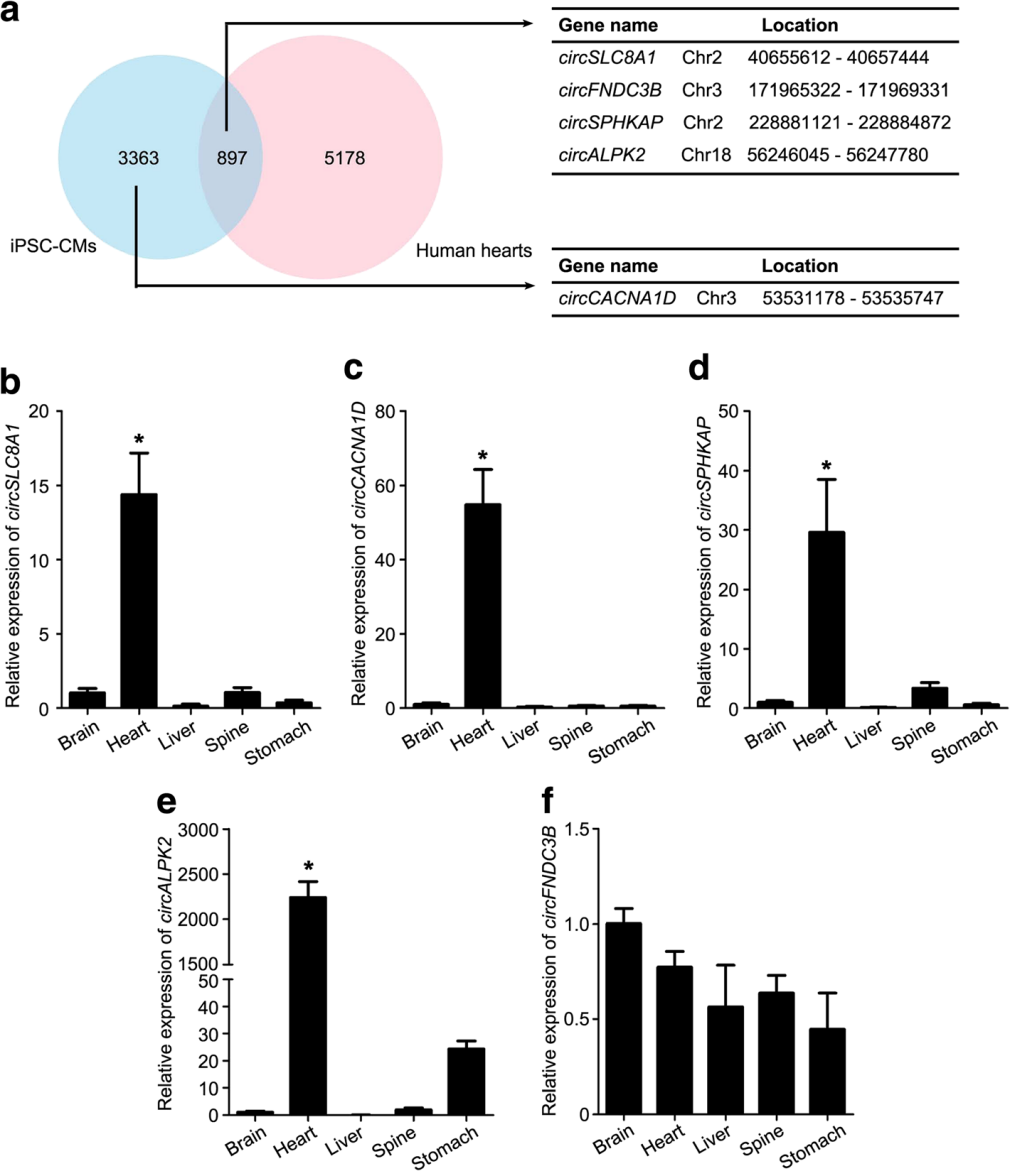
Differential expression of circRNAs in human tissues. **a** Comparison of our data with circRNAs identified in human tissues by Tan et al. [18] revealed a total of 897 overlapped circRNAs. Quantitative real-time PCR analysis showed significant upregulation of *circSLC8A1* (**b**), *circCACNA1D* (**c**), *circSPHKAP* (**d**) and *circALPK2* (**e**) transcripts in fetal heart, compared to other human tissues. **f** No significant change of *circFNDC3B* expression detected among these fetal tissues. Data shown as mean ± SD, *n* = 3; one-way ANOVA followed by Turkey’s test, *p < 0.05. CM cardiomyocyte, iPSC induced pluripotent stem cell

In order to seek more reliable cardiac circRNAs, a total of 6075 circRNAs from normal human hearts were compared with our 4260 circRNAs identified in hiPSC-CMs [18]. As shown in Fig. 5a, a total of 897 circRNAs were expressed in both hiPSC-CMs and human hearts, presented in Additional file 9: Table S5. However, there were also many hiPSC-CM or human tissue-specific circRNAs, which were probably contributed by the immature of hiPSC-CMs or heterogeneity of biopsies samples. While *circSLC8A1*, *circFNDC3B*, *circSPHKAP* and *circALPK2* were among the 897 overlapped circRNAs, *circCACNA1D* was only detected in our data.

We subsequently evaluated the expression of these five circRNAs in human fetal tissues. Four circRNAs (circ*SLC8A1*, *circCACNA1D*, *circSPHKAP* and *circALPK2*) displayed a significantly higher expression in fetal heart than other samples (Fig. 5b–e), while no obvious change of *circFNDC3B* expression was detected among different tissues (Fig. 5f).

**Fig. 6:**
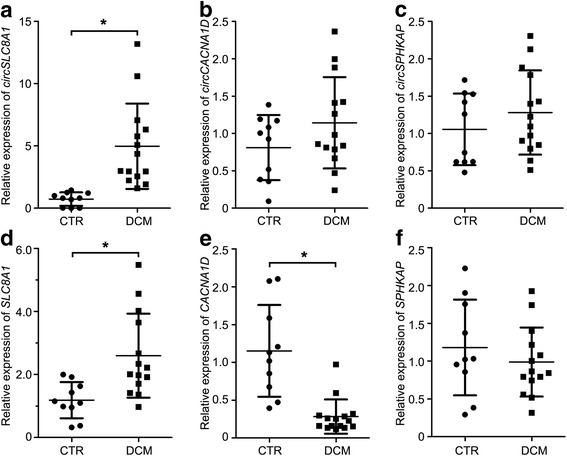
Expression of circRNAs in healthy and diseased human hearts. Quantitative real-time PCR analysis showed significant increased expression of *circSLC8A1* (**a**) in heart samples from patients with dilated cardiomyopathy (DCM), while no change of *circCACNA1D* (**b**) and *circSPHKAP* (**c**) expression was observed between control (CTR, *n* = 10) and DCM (*n* = 14) groups. Expression of *SLC8A1* (**d**), *CACNA1D* (**e**) and *SPHKAP* (**f**) mRNA also determined by qRT-PCR. Data shown as mean ± SD, Student’s *t* test, *p < 0.05

In order to assess the feasibility of using cardiac circRNAs as biomarkers for heart diseases, we performed qRT-PCR to compare the expression level of these circRNAs in heart tissues between healthy individuals and patients suffering from dilated cardiomyopathy (DCM). As a well-known cardiomyopathy marker, natriuretic peptide B (*NPPB*) expression significantly increased in the heart tissues from DCM patients, compared with that from healthy control (Additional file 10: Figure S4A). Among the five circRNAs, only *circSLC8A1* expression was significantly increased in the DCM group (Fig. 6 and Additional file 10: Figure S4).

We speculated that the upregulation of *circSLC8A1* in the DCM group might depend on the increase of its linear isoform. To verify our hypothesis, qPCR was performed to detect their linear transcripts, using specific primers. Consistent with our hypothesis, the linear transcripts of *SLC8A1* were also significantly increased in DCM samples when compared with the control group (Fig. 6d). Worthy of mention is that the average fold-change value of *circSLC8A1* expression was much higher than its corresponding linear transcripts (Fig. 6a, d), probably due to the higher stability of circRNAs.

Despite significantly decreased expression of its linear isoform in the DCM group, no notable difference of *circCACNA1D* expression was observed between healthy and DCM heart (Fig. 6b, e). Correspondingly, our previous bioinformatics analyses have identified *circCACNA1D* as one of the host gene-independent circRNAs (Additional file 8: Table S4).

In addition to their circular isoforms, no statistical difference of *SPHKAP*, *ALPK2* and *FNDC3B* mRNAs was observed between the normal and DCM groups (Fig. 6 and Additional file 10: Figure S4).

## Discussion

Pluripotent stem cells (PSCs) including ESCs and iPSCs hold great promise for stem cell therapy of heart diseases. However, understanding of the mechanisms of human heart development and cardiac differentiation is limited, which remains a significant barrier to developing effective approaches to generate homogeneous cardiomyocytes. Emerging evidence on cardiac-specific expression of some newfound circRNAs indicates that circRNAs might be also involved in heart development, and serve as biomarkers for differentiated CMs and heart diseases. Such hypothesis prompted us to perform comparative analyses of circRNA and mRNA profiles in hiPSCs and hiPSC-CMs. In this study, we totally identified 5602 circRNAs with at least two unique reads crossing the back-spliced junctions. Most of these circRNAs presented cell-specific expression in hiPSCs or hiPSC-CMs. Subsequent studies revealed an abundant expression of cardiac circRNAs, such as *circSLC8A1*, *circCACNA1D*, *circSPHKAP* and *circALPK2*, in heart tissues as well as hiPSC-derived CMs. Furthermore, the expression level of *circSLC8A1* significantly increased in specimens from patients with DCM when compared to the healthy controls.

We found that many highly expressed circRNAs in hiPSC-CMs were derived from cardiac genes, which were involved in heart development, membrane trafficking and muscle structure development. These circRNAs were also identified in hESC-derived CMs [18]. The most abundant circRNA in both hiPSC-CMs and hESC-CMs is circularized from the first exon of Solute Carrier Family 8 Member A1 (*SLC8A1*), a Na(+)–Ca(2+) exchanger localized at the cardiac sarcolemma and extruding Ca(2+) from the cell during excitation–contraction coupling. Although most circRNAs revealed a host gene-dependent expression, we still found some circRNAs showing a poor correlation with their relevant linear mRNAs in hiPSCs and hiPSC-CMs. This is probably because of the prolonged stability of RNAs by circularization, or a unique biogenesis mechanism of these circRNAs. Moreover, our analyses indicated that the proportion of such circRNAs significantly increased in hiPSC-CMs compared to that in hiPSCs.

One key discovery in our study is that circRNAs were enriched in differentiated CMs, rather than stem cells such as hiPSCs. While only a few back-splicing reads, accounting for 6% of total mapped reads, were identified in hiPSCs, the ratio of circRNA reads in hiPSC-CMs significantly increased to 17.5%. Consistently, recent studies have uncovered the enrichment of circRNAs in differentiated neurons and mammalian brain, while less abundant circRNAs were observed in proliferating colorectal cancer and gliomas compared to normal tissues [25–27]. These data suggest that differentiated cells might activate a different suit of genes to facilitate circRNA biogenesis and/or stability. In this regard, two RNA binding proteins have been reported to regulate circRNA formation. Quaking (QKI) is an alternative splicing factor that induces exon circularization via binding sites in flanking introns [28]. RNA binding Motif protein 20 (RBM20), whose loss causes DCM, has been showed to be crucial for circularization of the I-band region of the titin (*TTN*) transcript [16]. By RNA sequencing and qRT-PCR, we observed a cardiac-specific expression of *RBM20*, as well as a significant increase of *QKI* in hiPSC-CMs compared to hiPSCs. *QKI* knocking down in hiPSC-CMs obviously decreased the expression of circRNAs, including *circSLC8A1*, *circFNDC3B*, *circCACNA1D*, *circSPHKAP* and *circALPK2*, suggesting the critical roles of Quaking protein in circRNA generation. However, more regulatory factors accelerating circRNA expression in CMs remain to be identified in further studies.

Stability, sensitivity and specificity are considered essential features of a biomarker [29, 30]. Previous studies have revealed that circRNAs are more resistant to nucleases and thus have longer half-lives than corresponding linear RNAs [31, 32]. Combined data from Tan et al.’s study [18] and our study suggested a cell-specific expression of many circRNAs, such as *circSLC8A1*, *circCACNA1D*, *circSPHKAP* and *circALPK2*, in both hESC-derived and hiPSC-derived CMs. We further found that these four circRNAs are selectively abundant in fetal heart, rather than other tissues including the brain, liver, spine and stomach. Therefore, these cardiac circRNAs might serve as alternative biomarkers of cardiomyocytes.

Multiple circRNAs have been suggested as potential biomarkers of specific cancer diseases and CD28-related CD8(+) T-cell ageing [33–36]. Du et al. [37] found that *circFOXO3* was highly enriched in hearts from aged patients and mice, and promoted senescence by retaining anti-senescent and anti-stress proteins in the cytoplasm. In this study, we detected a significant increase of *circSLC8A1* in the DCM group compared to healthy controls. In Tan et al.’s study [18], the expression of *circSLC8A1* presented an upward trend in diseased hearts including DCM, HCM and IHD, in spite of lacking statistical significance. The lack of statistical difference in Tan et al.’s study may be due to a small sample size or the effects of outliers in the control group. Most recently, Siede et al. [19] also reported a significant upregulation of *circSLC8A1* in DCM biopsy material. Our study indicated a positive correlation of *circSLC8A1* expression with its linear transcript. However, the circular transcripts of *SLC8A1* seemed more stable than its linear transcript, as we detected a higher fold-change value of *circSLC8A1* expression than its corresponding linear transcripts. Taken together, the increased expression of *circSLC8A1* may be used to indicate the pathological status of heart disease. However, future studies are warranted to study the relevance of *circSCL8A1* and other heart diseases. Profiling circRNA expression in blood from healthy individuals or patients suffering heart disease would help to identify circulating circRNA biomarkers for clinical diagnosis [38].

## Conclusions

Our data revealed more enriched expression of circRNAs in differentiated CMs, detected a number of host gene-independent circRNAs and identified a number of cardiac-specific circRNAs, including *circSLC8A1*, *circCACNA1D*, *circSPHKAP* and *circALPK2*, which might be used as biomarkers. Furthermore, the disorder of *circSLC8A1* expression in heart tissue has been linked to the pathological status of heart diseases. More efforts are needed to uncover their roles in heart development and cardiovascular pathology, and to identify novel circRNA biomarkers in blood of patients.

## Additional files

Additional file 1: **Table S1.** Presenting primers used for circRNA detection in this study. (XLSX 10 kb)

Additional file 2: **Figure S1.** Showing characterization of human iPSCs and differentiated cardiomyocytes. (**A**) qRT-PCR detection of total and endogenous expression of reprogramming factors *OCT4* (also known as *POUF1*) and *SOX2*, as well as stem cell marker *NANOG* in fibroblasts, hiPSCs and hESCs. Data shown as mean ± SD (*n* = 3, one-way ANOVA followed by Turkey’s test, *p < 0.05). (**B**) Representative photographs of embryoid bodies (EBs) on days 2 and 6 of EB formation under bright field at 100× magnification. (**C**)–(**H**) Stage-specific expression of biomarker genes of early mesoderm (*BRA*), cardiogenic mesoderm (*MESP1*), cardiac-specific progenitors (*TBX5*) and structural genes (*TNNT2*, *MYH6* and *MYH7*), respectively, during cardiac differentiation from hiPSCs. Data shown as mean ± SD (*n* = 3, one-way ANOVA followed by Turkey’s test, *p < 0.05 compared to day 0 group). (PDF 382 kb)

Additional file 3: Video 1. Showing beating cardiomyocytes derived from hiPSCs.

Additional file 4: **Table S2.** Presenting circRNAs identified in hiPSCs and hiPSC-CMs (XLSX 315 kb)

Additional file 5: **Figure S2.** Showing characterization of circRNAs by Sanger sequencing. Schematic diagrams of circularization of *SLC8A1* (**A**), *ALPK2* (**B**) and *SPHKAP* (**C**) transcripts by back-splicing of exons. Back-splicing sites of these three circRNAs verified by Sanger sequencing (PDF 443 kb)

Additional file 6: **Figure S3.** Showing digital mRNA expression from RNA-sequencing. (**A**) Hierarchical clustering (heat map) showed differential gene expression among hiPSCs (iPSCs) and hiPSC-CMs (CMs). (**B**) Scatter plot showed differentially expressed mRNAs with at least 2-fold change between hiPSCs and hiPSC-CMs. Upregulated genes in hiPSCs indicated as green dots, pink dots refer to genes increased in hiPSC-CMs. GO analyses of upregulated genes in hiPSCs (**C**) and hiPSC-CMs (**D**) revealed obvious change of biological processes between hiPSCs and their differentiated CMs. (**E**), (**F**) Representative upregulated genes in hiPSCs or hiPSC-CMs, respectively, involved in cell-specific biological processes (PDF 506 kb)

Additional file 7: **Table S3**. Presenting differently expressed genes with at least 2-fold change in hiPSCs and hiPSC-CMs (XLSX 83 kb)

Additional file 8: **Table S4**. Presenting circRNAs exhibiting distinguishing expression pattern with their linear parental transcripts in hiPSCs and hiPSC-CMs (XLSX 18 kb)

Additional file 9: **Table S5**. Presenting circRNAs detected in both hiPSC-CMs and human heart tissues (XLSX 40 kb)

Additional file 10: **Figure S4**. Showing expression of selected circRNAs in healthy and diseased human hearts. Quantitative real-time PCR analysis showed increased expression of *NPPB* (**A**), marker of cardiomyopathy, in heart samples from DCM patients. No change of *circALPK2* (**B**) and *circFNDC3B* (**D**), as well as their mRNA expression (**C**), (**E**), observed between the control (CTR, *n* = 10) and DCM (*n* = 14) groups. Data shown as mean ± SD, Student’s *t* test, *p < 0.05 (PDF 256 kb)

## Abbreviations

circRNA: Circular RNA
DCM: Dilated cardiomyopathy
DMEM: Dulbecco’s modified Eagle medium
EB: Embryoid body
ESC: Embryonic stem cell
iPSC: Induced pluripotent stem cell
iPSC-CM: iPSC-derived cardiomyocyte
KSR: KnockOut Serum Replacement
ncRNA: Noncoding RNA
PSC: Pluripotent stem cell
qRT-PCR: Quantitative real-time polymerase chain reaction
