# Supplementary figures and images for: Signature of circular RNAs in human induced pluripotent stem cells and derived cardiomyocytes
Source: Stem Cell Res Ther. 2018 Mar 9;9:56. doi: 10.1186/s13287-018-0793-5 (PMC5845222; doi:10.1186/s13287-018-0793-5)

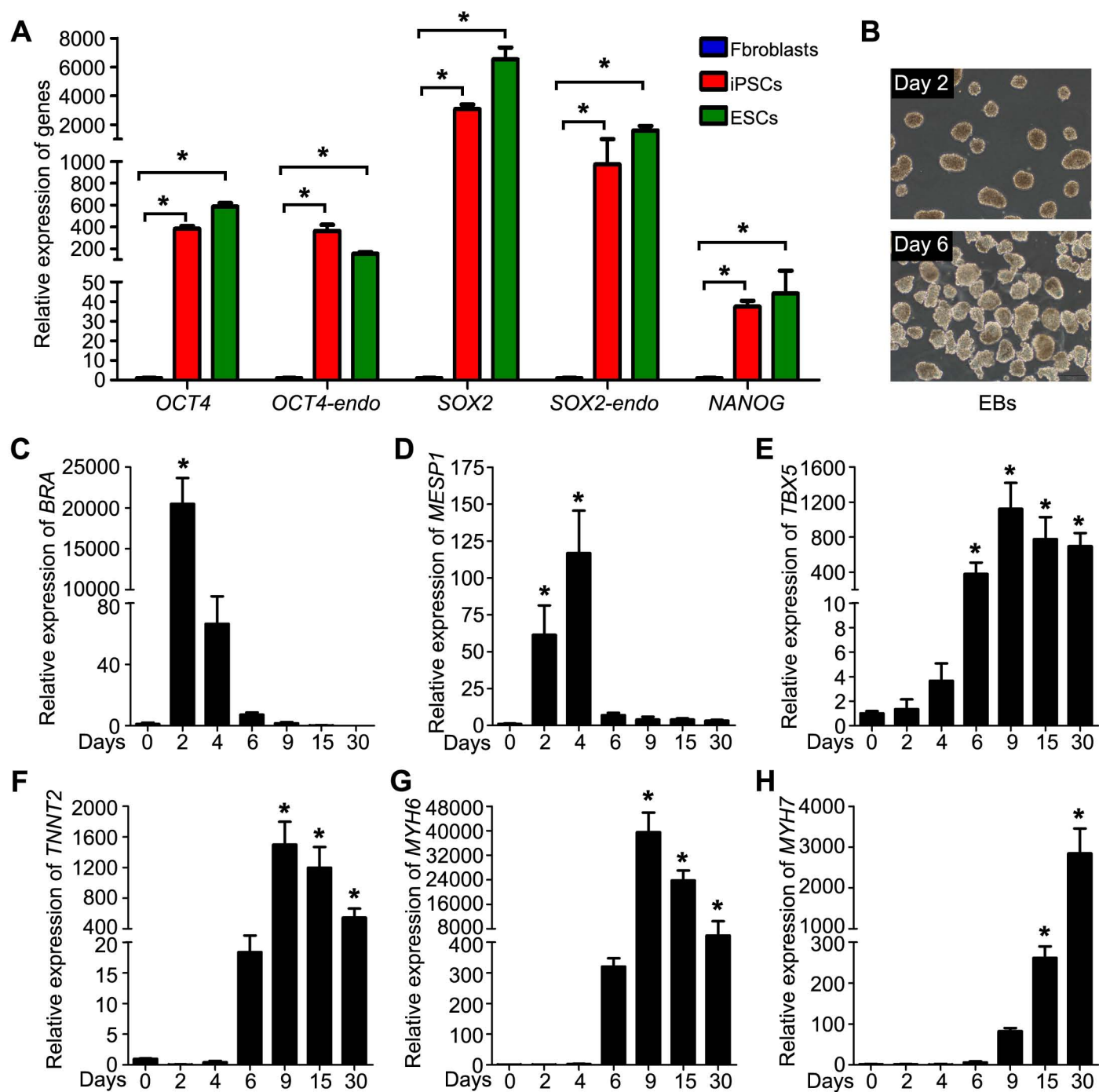

Supplemental figure 1

Supplement: Supplementary file 2 — Figure S1. Showing characterization of human iPSCs and differentiated cardiomyocytes. (A) qRT-PCR detection of total and endogenous expression of reprogramming factors OCT4 (also known as POUF1) and SOX2, as well as stem cell marker NANOG in fibroblasts, hiPSCs and hESCs. Data shown as mean ± SD (n = 3, one-way ANOVA followed by Turkey’s test, *p < 0.05). (B) Representative photographs of embryoid bodies (EBs) on days 2 and 6 of EB formation under bright field at 100× magnification. (C)–(H) Stage-specific expression of biomarker genes of early mesoderm (BRA), cardiogenic mesoderm (MESP1), cardiac-specific progenitors (TBX5) and structural genes (TNNT2, MYH6 and MYH7), respectively, during cardiac differentiation from hiPSCs. Data shown as mean ± SD (n = 3, one-way ANOVA followed by Turkey’s test, *p < 0.05 compared to day 0 group). (PDF 382 kb) [file 13287_2018_793_MOESM2_ESM.pdf]

## Slide 1
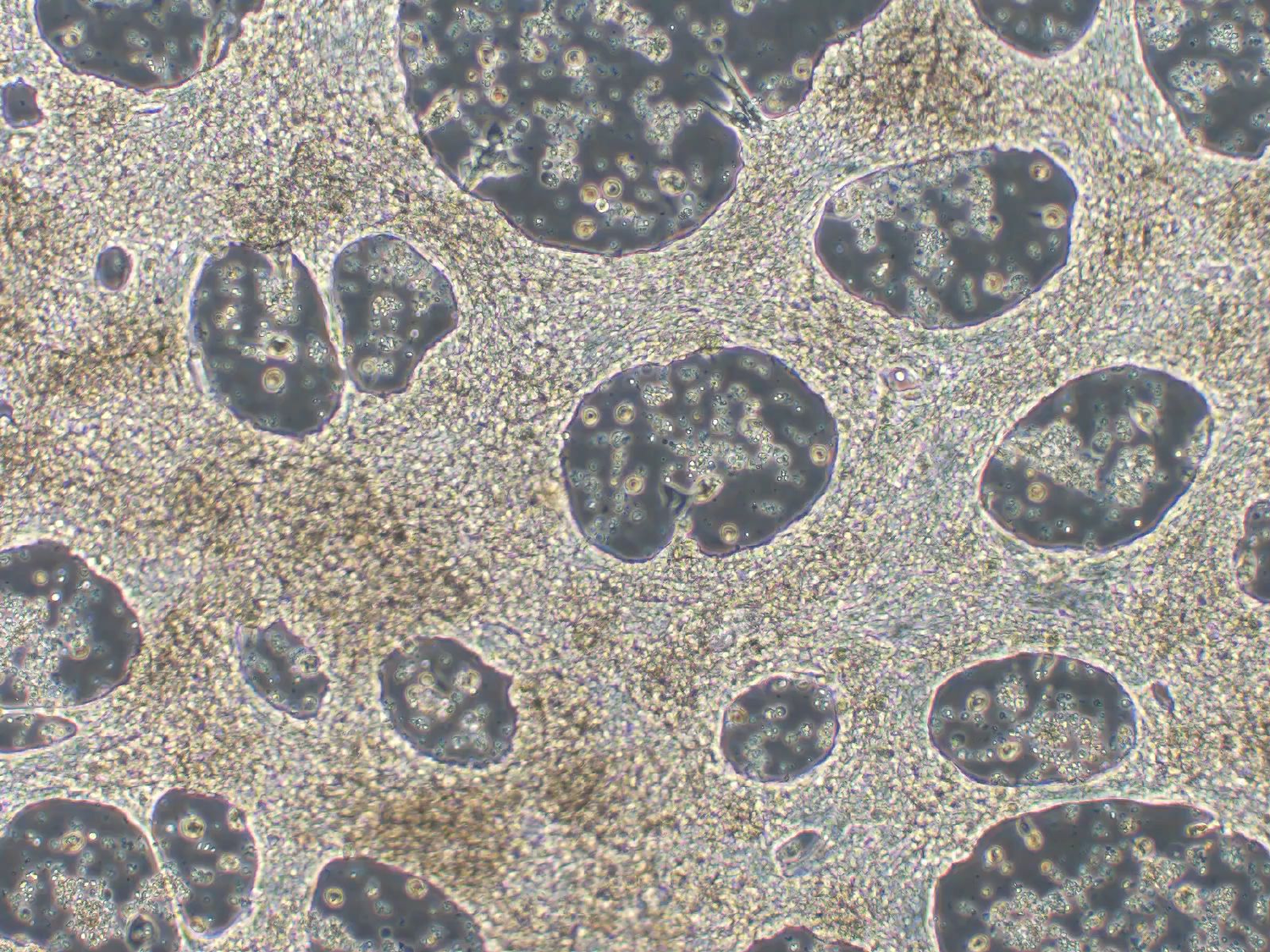

Supplement: Supplementary file 3 — Video 1. Showing beating cardiomyocytes derived from hiPSCs. [file 13287_2018_793_MOESM3_ESM.pptx]

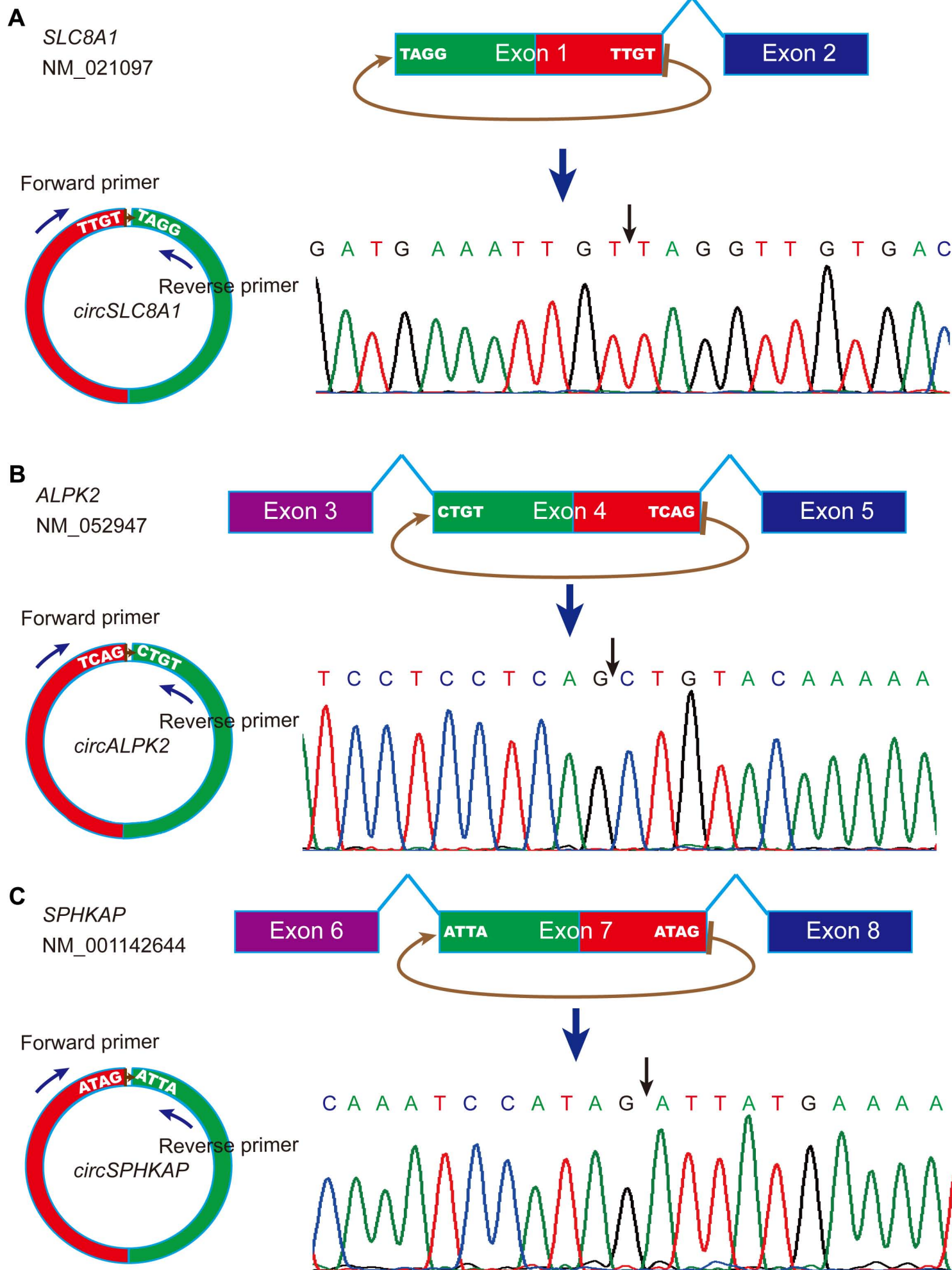

Supplemental figure 2

Supplement: Supplementary file 5 — Figure S2. Showing characterization of circRNAs by Sanger sequencing. Schematic diagrams of circularization of SLC8A1 (A), ALPK2 (B) and SPHKAP (C) transcripts by back-splicing of exons. Back-splicing sites of these three circRNAs verified by Sanger sequencing (PDF 443 kb) [file 13287_2018_793_MOESM5_ESM.pdf]

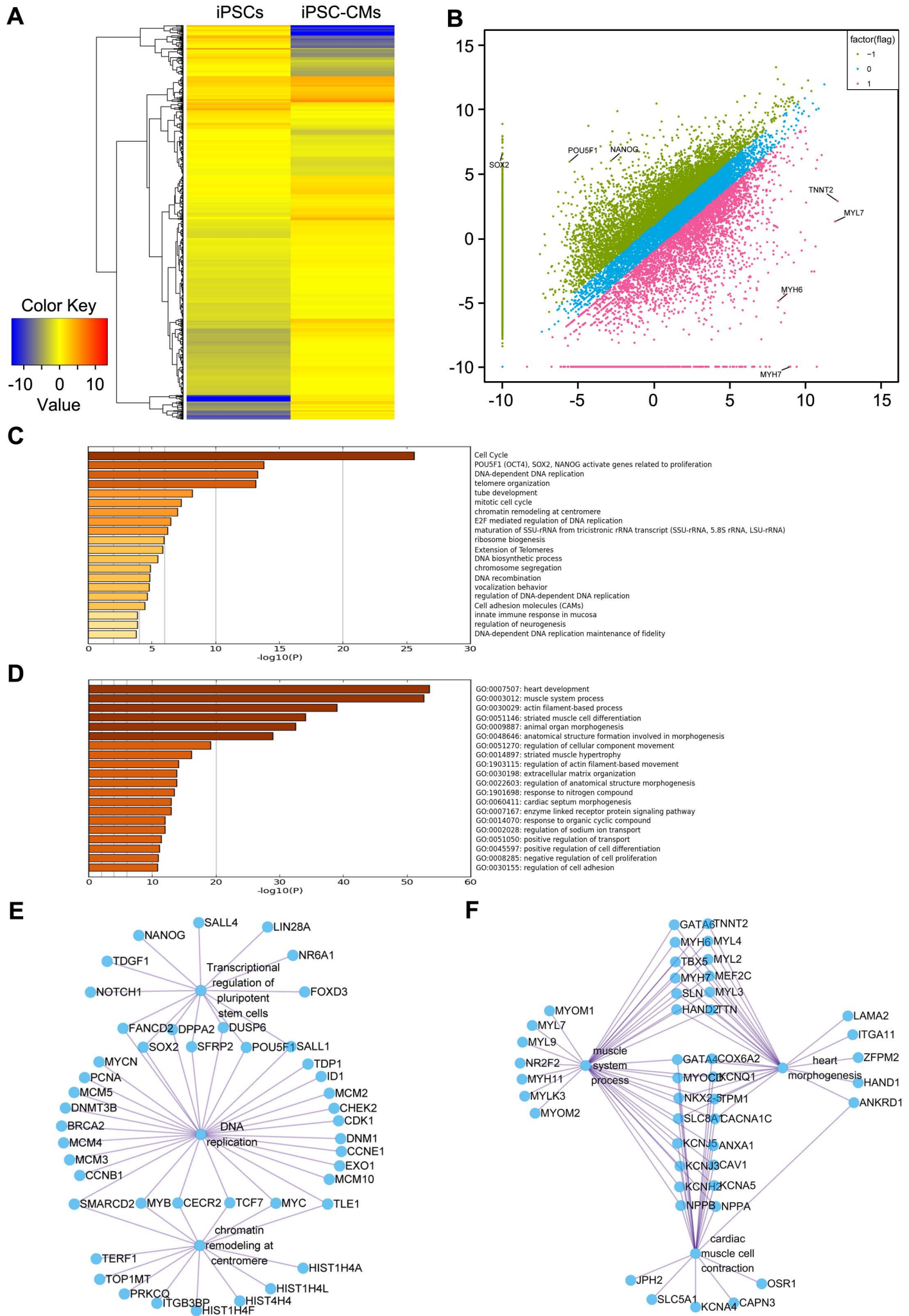

Supplemental figure 3

Supplement: Supplementary file 6 — Figure S3. Showing digital mRNA expression from RNA-sequencing. (A) Hierarchical clustering (heat map) showed differential gene expression among hiPSCs (iPSCs) and hiPSC-CMs (CMs). (B) Scatter plot showed differentially expressed mRNAs with at least 2-fold change between hiPSCs and hiPSC-CMs. Upregulated genes in hiPSCs indicated as green dots, pink dots refer to genes increased in hiPSC-CMs. GO analyses of upregulated genes in hiPSCs (C) and hiPSC-CMs (D) revealed obvious change of biological processes between hiPSCs and their differentiated CMs. (E), (F) Representative upregulated genes in hiPSCs or hiPSC-CMs, respectively, involved in cell-specific biological processes (PDF 506 kb) [file 13287_2018_793_MOESM6_ESM.pdf]

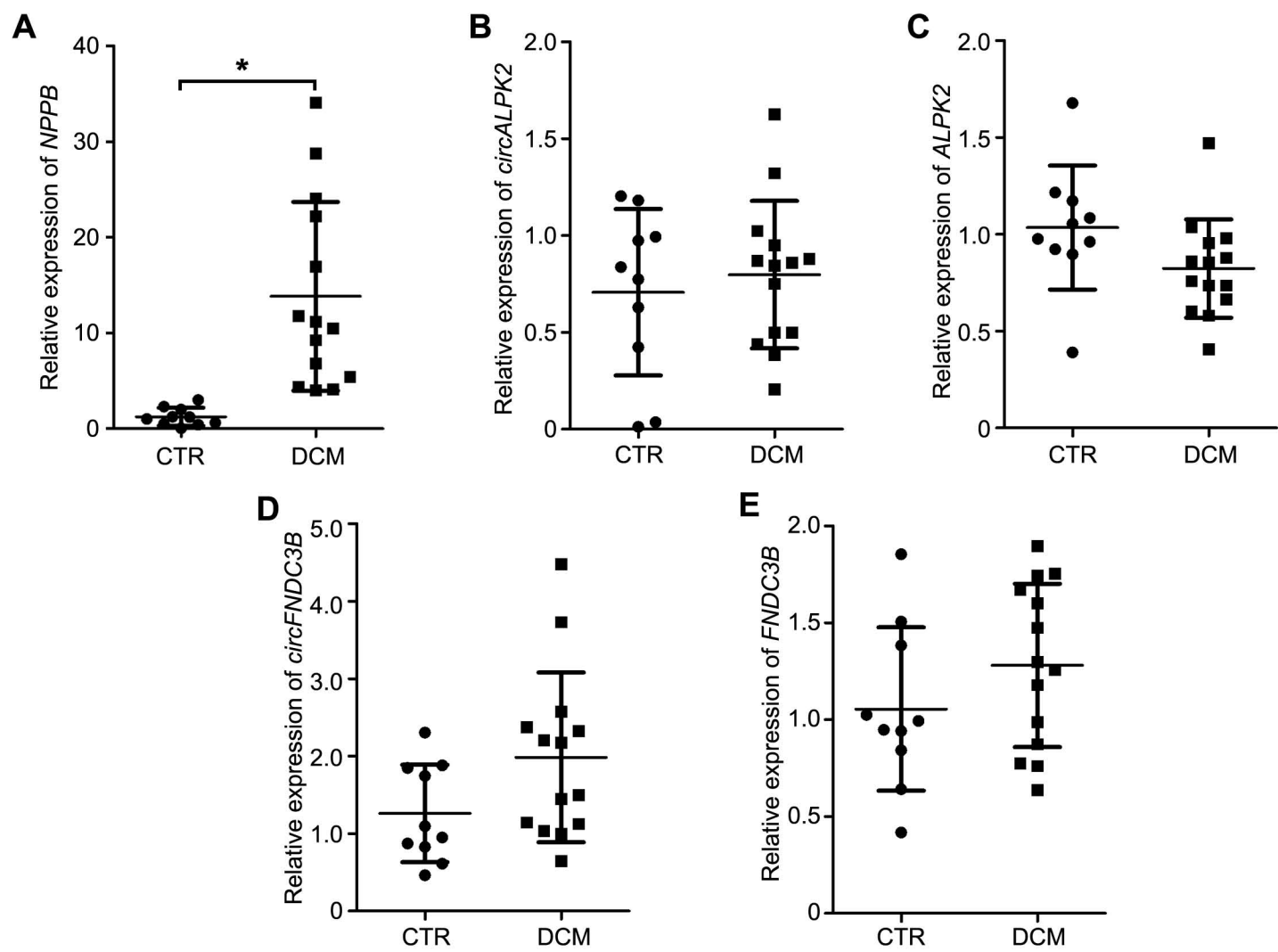

Supplemental figure 4

Supplement: Supplementary file 10 — Figure S4. Showing expression of selected circRNAs in healthy and diseased human hearts. Quantitative real-time PCR analysis showed increased expression of NPPB (A), marker of cardiomyopathy, in heart samples from DCM patients. No change of circALPK2 (B) and circFNDC3B (D), as well as their mRNA expression (C), (E), observed between the control (CTR, n = 10) and DCM (n = 14) groups. Data shown as mean ± SD, Student’s t test, *p < 0.05 (PDF 256 kb) [file 13287_2018_793_MOESM10_ESM.pdf]
